# Differences in quality of life among college student electronic cigarette users

**DOI:** 10.3934/publichealth.2018.4.454

**Published:** 2018-12-03

**Authors:** S. Lee Ridner, Rachel J. Keith, Kandi L. Walker, Joy L. Hart, Karen S. Newton, Timothy N. Crawford

**Affiliations:** 1 College of Nursing, East Tennessee State University, Johnson City, TN, USA; 2 School of Medicine, University of Louisville, Louisville, KY, USA; 3 Department of Communication, University of Louisville, Louisville, KY USA; 4 Campus Health Services, University of Louisville, Louisville, KY USA; 5 Department of Population and Public Health Sciences, Wright State University, Dayton, OH, USA

**Keywords:** electronic cigarette, e-cigarette, college students, tobacco use, quality of life

## Abstract

**Purpose:**

The purpose of this study was to explore an association between e-cigarette use and Quality of Life (QOL) among college students.

**Methods:**

During February 2016, 1,132 students completed an online survey that included measures of tobacco use and the WHOQOL-BREF instrument. Differences were tested using Chi-square, Fisher's exact test, and ANOVA, and regression was used to assess possible relationships.

**Results:**

E-cigarettes were used by 6.97% of the participants, either solo or along with traditional cigarettes. Bivariate analyses suggest that male college students are more likely than females to use e-cigarettes, either solo or in combination with traditional cigarettes (χ^2^ =19.4, *P* < .01). Lesbian, gay, and bisexual students are more likely than heterosexual students to use traditional cigarettes, either solo or in combination with e-cigarettes (χ^2^ = 32.9, *P* < .01). Multivariate models suggest that for every 10-unit increase in overall QOL, psychological well-being, social relations or environmental health the adjusted odds of being a sole cigarette user were significantly lower (all, *P* < .01), respectively. For every 10-unit increase in psychological well-being the adjusted odds of being a dual user was significantly lower (OR = .83, *P* = .026).

**Conclusions:**

Findings indicate that lower quality of life appears to be connected to tobacco use.

## 1. Introduction

The electronic cigarette (e-cigarette) was first introduced a decade ago.[1] Across time, the popularity and design of these devices have changed remarkably. The first device contained a small cartridge of nicotine solution, resembled a cigarette, and was promoted as a smoking cessation tool. Today e-cigarettes are modifiable, with users able to personalize the amount of nicotine (if any) and the flavor of the solution.[2] Paralleling these changes in technology has been an increase in the number of people using e-cigarettes.

Between 2011 and 2017, current e-cigarette use rose from 1.5% to 11.7% among U.S. high school students.[3] In 2014, 3.7% of U.S. adults reported current e-cigarette use.[4] Use among young adults 18 to 24 years of age is higher than in the adult general population; 13.6% reported using the devices every day, some days, or occasionally.[5]

A similar trend has been noted among college students. A 2009 random sample of North Carolina college students showed that 4.9% of students were “ever” users and 1.5% had used e-cigarettes in the past month.[6] Of more than 95,000 randomly selected college students on 137 campuses in 2016, 14.8% were “ever” users and 3.7% had used in the last 30 days.[7] It is important to note that nearly 20% of the respondents in that sample were 25 years or older. Among college students, increased use may be related to them being more likely to accept the use of e-cigarettes in public compared to using traditional cigarettes in public.[8] Other studies suggest that the greater information exposure to e-cigarettes and alternative tobacco products puts college students at greater risk for e-cigarette use.[9]

A number of studies have found lower levels of well-being among college students who use tobacco when compared to non-tobacco users.[10],[11] Well-being is a multidimensional construct that is conceptualized in a number of different ways. It may include psychological components, positive and negative affect, satisfaction with life, and overall happiness.[12]–[14] Others assert that quality of life (QOL) is a subjective sense of well-being that includes multiple dimensions.[15] No matter the conceptual approach used to determine QOL or well-being, an evaluation of one's life is required.

QOL related to smoking and disease states among smokers has been examined in a number of cross-sectional and cohort studies. See Goldenberg, Danovitch, and IsHak (2014) for a review.[16] In general, smoking status and the failure to quit smoking are associated with lower levels of QOL. One study in that review found lower levels of QOL among hookah users.[17] That study was conducted with middle-aged adults (mean 42.1 years) in a Middle Eastern country.

Although there have been a number of studies examining the relationship between QOL and smoking, there's a paucity of research examining QOL and smoking among college students.[18]–[20] Extant studies focus on QOL as it relates to specific diseases (e.g. asthma), disabilities, or stressful life events. None have examined QOL among college students who use e-cigarettes. Therefore, the purpose of this study was to explore differences in QOL among college students who use and do not use e-cigarettes. The specific aim of this study was to examine associations between QOL and e-cigarette use among college students.

## 2. Materials and Methods

### 2.1. Participants

A random sample of 5,000 undergraduate students, attending a university in the southeastern United States who were 18 to 26 years of age, received an electronic invitation to participate in an anonymous online survey focused on well-being from campus health promotion services. Students who completed the survey had the option to enter a lottery for prizes. The prizes included massages, a Fitbit, Google Chrome Cast, Roku, an iPad, and a bicycle. The student body was comprised of nearly 15,000 undergraduates, 50% were female, and 73% were white. The final sample consisted of 1,132 students (23% response rate). All data were de-identified. This study is a secondary analysis of cross-sectional data collected for program evaluation in February 2016; the University of Louisville Institutional Review Board reviewed and approved this study.

### 2.2. Measures

The students completed the World Health Organization Quality of Life–BREF survey.[21],[22] The survey is an abbreviated quality of life (QOL) measure that contains 26 items from the original 100–item survey.[22] The original version had 24 facets of QOL and the BREF contains an item assessing each facet. A 5-point Likert-type scale is used and depending on the item, responses range from “very poor” to “very good”, “very dissatisfied” to “very satisfied”, “not at all” to “an extreme amount”, “not at all” to “completely”, and “never” to “always”. The QOL measure includes physical health (7 items), psychological (6 items), social relationships (3 items), and environmental (8 items) domains. Three items on the scale are reverse scored. The raw data are transformed to a 0 to 100 scale using a standard procedure outlined by WHO. Higher scores on the scale reflect higher levels of QOL. Mean scores for the domains among international college students range from 63.43 to 70.63 for physical health, 62.58 to 64.24 for psychological, 63.41 to 67.27 for social relationships, and 52.3 to 57.6 for environmental.[23],[24] Cronbach's alphas for the domains among U.S. college students ranged from .70 to .75 in one study[25] and .73 to .86 in another.[26] There is one additional item that assesses overall QOL, “how would you rate your quality of life?” and one item that assesses health, “how satisfied are you with your health?” Both items are scored using a 5-point Likert-type scale that can be transformed to a 0 to 100 scale.

Students self-reported their age, sex, sexual identity (lesbian, gay bisexual [LGB]), year in school, grade point average, and race. The seven categories for race (American Indian/Alaskan Native, Asian, Black/African American, Hispanic/Latino, Pacific Islander, Two or More Races, and White) were collapsed into two categories (White or Non-White). Tobacco use was assessed by asking students to self-report how many days in the past 30 they used: 1) traditional cigarettes and 2) e-cigarettes. Responses included “never used”, “0 days”, “1-2 days”, “3-5 days”, “6-9 days”, “10-19 days”, “20-29 days”, and “all 30 days”. The data were dichotomized to reflect any use in the past 30 days. The main outcome variable (tobacco use) was categorized into non-users, dual users (i.e., used both traditional cigarettes and e-cigarettes), sole e-cigarette users, and sole traditional cigarette users.

### 2.3. Statistical Analysis

Study participant characteristics were expressed as frequency (%) for categorical variables. Associations between demographic characteristics and tobacco use were examined using Chi-square tests, Fisher's exact test, or ANOVA as appropriate for the level of data. Multivariate logistic regression models were conducted to examine the relationship between tobacco use and QOL. For each model, tobacco non-users were the reference group. Demographic characteristics that were significantly associated with tobacco use were included in the models. Models were adjusted for sex, race, and sexual identity. Data were analyzed using SAS, version 9.4 (SAS Institute, Inc., Cary, North Carolina) and *P*-values <.05 were regarded as statistically significant.

## 3. Results

### 3.1. Participants

Summary statistics of the study sample appear in Table 1. The majority of participants were female (62%), white (78%), and heterosexual (87%). Participants were relatively mixed by academic level. The average age of participants was 21.2 years (*SD* = 1.8) and the average GPA was 3.05 (*SD* = 0.86). When compared to the university population, females and whites were overrepresented and freshmen were underrepresented in the sample. The QOL scores were as follows: overall QOL (*M* = 71.2, *SD* = 17.7), physical health (*M* = 71.1, *SD* = 14.5), psychological well-being (*M* = 63.0, *SD* = 17.4), social relations (*M* = 64.9, SD = 20.9), and environmental health (*M* = 67.1, *SD* = 14.8).

**Table 1.** Demographic Characteristics and the Comparison of Tobacco Use/Non-Use (N=1,132).

		Current Tobacco Use				
		Entire Sample N=1132 (%)	Non-Use n=947 (%)	Dual Use n=42 (%)	Sole E-Cigarette n=37 (%)	Sole Cigarette n=106 (%)
Age - mean (sd)		21.2 (1.8)	21.2	21.6	21.4	21.3
GPA - mean (sd)		3.05 (.86)	3.1	3.2	2.8	3.0
Sex*	Female	706 (63)	608 (64)	16 (38)	15 (41)	67 (63)
	Male	426 (37)	339 (36)	26 (62)	22 (59)	39 (37)
Race	White	922 (78)	773 (82)	31 (74)	31 (84)	87 (82)
	Non-White	250 (22)	214 (18)	11 (26)	6 (16)	19 (18)
Sexual Identity*	Heterosexual	984 (87)	845 (89)	31 (74)	32 (86)	76 (72)
	LGB	147 (13)	101 (11)	11 (26)	5 (14)	30 (28)
Academic Level	Freshman	205 (18)	169 (18)	4 (9)	8 (22)	24 (23)
	Sophomore	292 (26)	249 (26)	12 (29)	6 (16)	25 (24)
	Junior	267 (24)	233 (25)	10 (24)	8 (22)	16 (15)
	Senior	368 (32)	296 (31)	16 (38)	15(40)	41 (38)
WHOQOL-BREF Scores						
Quality of Life–mean (sd)*		71.2 (17.7)	72.2	67.6	68.9	64.6 ^a^
Physical Health-mean (sd)		71.1 (14.5)	71.7	67.7	71.7	67.3
Psychological-mean (sd) *		63.0 (17.4)	64.2	57.1	60.8	55.5 ^a^
Social Relations-mean (sd)*		64.9 (20.9)	66.7	63.5	65.8	59.0 ^a^
Environmental-mean (sd)*		67.1 (14.8)	67.7	63.0	67.5	63.2 ^a^

*Note.* ^a^ Post hoc difference from nonusers at *P* < .05. *P < .01

### 3.2. Characteristics of tobacco use

Study results based on tobacco use appear in Table 1. Of the 1,132 participants, 947 were non-tobacco users, 42 used both traditional cigarettes and e-cigarettes (dual use), 37 used only e-cigarettes (sole e-cigarette users), and 106 used only traditional cigarettes (sole traditional cigarette users). E-cigarettes were used by 6.97% of the participants, either solo or along with traditional cigarettes. There was no difference in tobacco use by age, academic level, or GPA between the groups. Male college students were more likely than females to use e-cigarettes, either solo or in combination with traditional cigarettes (χ^2^ = 19.4, *P* < .01). Lesbian, gay, and bisexual students are more likely than heterosexual students to use traditional cigarettes, either solo or in combination with e-cigarettes (χ^2^ = 32.9, *P* < .01). There were significant associations between tobacco use and overall QOL, physical health, psychological well-being, social relations, and environmental health (all, *P* < .01). Post-hoc analyses showed significant differences in these scores were found between non-users and sole-cigarette users (all, *P* < .05).

### 3.3. Quality of life and tobacco use

Table 2 provides the results of the multivariate logistic models examining the relationship between tobacco use patterns and quality of life. The Hosmer and Lemeshow tests for the models were non-significant (*P* = .10 – *P* = .56) indicating the models fit the data. After adjusting for race, sex, and sexual identity, overall QOL, physical health, social well-being, and environmental health scores were not associated with being a dual or sole e-cigarette user. However, as psychological well-being increased by 10-units, the odds of being a dual user compared to a non-user decreased (Odds Ratio (OR) = 0.83; 95% Confidence Interval (CI) [0.70, 0.98]). For every 10-unit increase in overall QOL, physical health, psychological well-being, or social relations the adjusted odds of being a sole cigarette user compared to a non-user were significantly lower (*OR* = .83, 95% CI [.74, .92], *P* < .001); (*OR* = .86, 95% CI [.75, .99], *P* = .029); (*OR* = .80, 95% CI [.72, .90], *P* < .001); (*OR* = .87, 95% CI [.79, .96], *P* = .003), and (*OR* = .84, 95% CI [.74, .96], *P* = .01), respectively. After adjusting for race, sex, and sexual identity there was no association between dual vs. sole cigarette user, dual vs. sole e-cigarette user, and sole e-cigarette vs. sole cigarette user and any of the QOL measures.

**Table 2.** Tobacco Use and Quality of Life (N=1,132).

	Dual Use			Sole E-cig Use			Sole Cigarette Use		
	OR	95% CI	*P*	OR	95% CI	*P*	OR	95% CI	*P*
QOL	0.88	[0.74, 1.04]	.135	0.90	[0.75, 1.08]	.244	0.83	[0.74, 0.92]	<.001
Physical health	0.84	[0.68, 1.03]	.097	1.00	[0.79, 1.26]	.971	0.86	[0.75, 0.99]	.029
Psychological well-being	0.83	[0.70, 0.98]	.026	0.90	[0.74, 1.08]	.239	0.80	[0.72, 0.90]	<.001
Social relations	0.97	[0.84, 1.12]	.688	1.00	[0.86, 1.17]	.997	0.87	[0.79, 0.96]	.003
Environmentalhealth	0.83	[0.69, 1.02]	.072	1.00	[0.80, 1.24]	.988	0.84	[0.74, 0.96]	.010

*Note.* Logistic regression adjusted for race, sex, sexual identity. Represents odds ratio of being in each category compared to non-users per 10-unit increase on WHOQOL-BREF scale.

## 4. Discussion

The purpose of this study was to examine associations between QOL and tobacco use patterns among college students. Interestingly, our findings suggest that as college students' perceived quality of life increases the likelihood that they will consume traditional cigarettes decreases. For dual use, psychological well-being appears to play a key role; that is, as psychological well-being increases likelihood of engaging in dual use decreases. As with traditional cigarette use, e-cigarette use appears to be connected to psychological distress.[27] Additional research is needed to further examine potential relationships between well-being and traditional cigarette and e-cigarette use and, when such relationships exist, to explicate their causes.

Although the percentage of e-cigarette users in our sample (6.97%) was comparable to the number of e-cigarette users nationally (4.1%) during Spring 2016,[7] the high overall rate of tobacco use is a cause for concern. When use rates for all tobacco products examined (i.e., sole cigarette use, sole e-cigarette use, and dual use) are combined, our results show that over 16% of study participants consumed one or more forms of tobacco. Additional work to prevent youth and young adult tobacco use as well as to encourage quitting is needed. Tobacco takes a heavy toll on young users—in terms of the likelihood of continued use as well as the cumulative health effects of such use. Given the interest of college students in newer and novel tobacco products, such as e-cigarettes, these populations seem especially important ones to target with prevention and cessation messaging.

Although female sole cigarette users outnumbered male sole cigarette users and the number of female tobacco users outnumbered male tobacco users, males were more frequently sole e-cigarette users and dual users. This finding, especially when combined with similar findings in previous studies,[28] suggests that because male college students are more likely to use e-cigarettes they are more at risk of potential health effects associated with such consumption. Although exact health effects are not yet clear, emerging findings point to several potential dangers.[28]–[30] Further, some studies have indicated that dual users may consume more nicotine or report greater withdrawal than sole e-cigarette or sole cigarette users;[31],[32] thus, future studies should examine overall levels of nicotine consumption by college males.

Previous work with sexual minorities has reported high levels of smoking,[33]–[36] and more recent studies indicate that use of both traditional cigarettes and e-cigarettes tends to be high in LGB populations.[37],[38] Our results also indicate a propensity among LGB students to smoke traditional cigarettes, either through sole use or in combination with e-cigarettes. Given the increased likelihood that LGB individuals will consume traditional and e-cigarettes, future research should seek to better understand the drivers for such use,[39]–[41] especially for adopting e-cigarette or other novel tobacco product use. In addition, health communication campaigns, such as the FDA's “This Free Life”, which targets young LGB adults, should attempt to reduce the tobacco burden in these communities.

## 5. Limitations

Despite interesting findings that contribute to the literature on college students' QOL perceptions and e-cigarette use, this study has several limitations. First, all data were self-reported and thus subject to the potential of associated biases (e.g., memory, mood). Second, although our study had similar percentages of e-cigarette users to other studies with college students, our findings were shaped by the questions asked and the study design did not allow for more in-depth responses to glean a richer understanding from participants who used e-cigarettes, either in a sole or dual capacity. Third, despite a response rate similar to other surveys of this type,[7] differences may exist between participants who chose to respond to the survey invitation and individuals who declined. Fourth, our survey was conducted on one campus and may not represent the views of all college students.

## 6. Conclusions

In spite of these limitations, our results support previous findings, extend past work in new ways, and point to future research directions. First, male college students are more likely to use e-cigarettes, either solo or in combination with traditional cigarettes. These use patterns, in our study as well as previous studies, suggest that targeted health messaging is needed for this population to raise awareness of (1) scientific uncertainty surrounding e-cigarette safety as well as (2) the likelihood of nicotine increases with dual use and associated dangers of such consumption. Second, LGB students are more likely to use traditional cigarettes, either solo or in combination with e-cigarettes. This finding, especially when combined with similar findings in past studies, indicates that health communication campaigns on campuses are needed for LGB college students. The dangers of traditional cigarettes are well documented and combining their use with newer tobacco products, such as e-cigarettes, has the potential to exacerbate negative health effects. Third, this study shows interesting links between perceived quality of life and use of tobacco products. In particular, as with consumption of traditional cigarettes,[27] psychological distress appears to be connected to use of e-cigarettes. Further, our findings suggest increases in psychological well-being result in decreased likelihood of dual use. The results of this study, one of the first to examine QOL and e-cigarette use, call for additional inquiry to more fully understand linkages in overall well-being and tobacco product consumption, especially with newer tobacco products, such as e-cigarettes. Fourth, both the e-cigarette use rate and overall patterns of tobacco consumption in our study point out that much work remains to be done on college campuses to lessen the likelihood that college students will begin using tobacco products and to promote cessation among students who are tobacco users. The results of this study lay important groundwork for future health prevention measures directed to groups most likely to use e-cigarettes and for future inquiries into the role that perceived QOL may play in tobacco initiation and continued use.
